# Comment on “Structural Factors That Determine the Amylolytic Properties of Starch–Lipid Complexes”

**DOI:** 10.1021/acs.jafc.4c12066

**Published:** 2025-07-29

**Authors:** Maame Esi Baidoo, Jonghoon Kang

**Affiliations:** Department of Biology, 15614 Valdosta State University, Valdosta, Georgia 31698, United States

In a recent publication in the *Journal of Agricultural and Food Chemistry*, Wang et al.^1^ showed that the preparation of starch–lipid complexes with predictable digestibility is possible with the selection of the appropriate complexing conditions of temperature and time. The authors prepared various starch–lipid complexes using debranched high-amylose maize starch (DHAMS) together with four different fatty acids (FAs) under different complexing conditions and determined their *in vitro* digestibility. Some of the properties discussed in this paper are the thermal properties of the DHAMS–FA complexes. They observed two thermal peaks in their differential scanning calorimetry (DSC) curves, which they classified as peak I and peak II for each of the DHAMS–FA complexes, which were due to their crystalline nature, with those forming V_I_ (type I) crystalline forms showing a peak for peak I and those forming V_II_ (type II) crystalline forms showing a peak for peak II. They explained the stability of the DHAMS–FA complexes in terms of the melting temperature (*T*_m_) and enthalpy change (Δ*H*), finding that the longer the chain length of fatty acids, the greater the stability of starch–FA complexes, and complexes prepared at higher complexing temperatures were more stable and therefore less susceptible to amylolysis. However, the paper does not address another essential thermodynamic parameter, entropy change (Δ*S*), which is important for understanding the mixing of biopolymers^2^ and the formation and melting of crystallites in starch–lipid complexes.^3^ Here, we examine their findings using statistical analyses to further explain their results, including finding the numerical values of Δ*S* in the melting of the DHAMS–FA complexes, their correlation with Δ*H*, and the potential implications of the relationship between Δ*H* and Δ*S* in the melting of starch–lipid complexes.

Since the process by which the DSC curves were obtained is a phase transition process characterized by the melting of the starch–lipid complex, Δ*S* can be computed using the following equation

1 $$\Delta S=\frac{\Delta H}{T_{m}}$$

where *T*_m_ is the melting temperature in kelvin.^4^ To compute Δ*S*, the values of Δ*H* and *T*_m_ were obtained from ref 1 for each of the DHAMS–FA complexes for both peak I (type I crystallites) and peak II (type II crystallites). A total of eight samples containing type I crystallites and 11 samples containing type II crystallites were obtained (Table 1). A linear regression using eq 2 shows a highly significant correlation between Δ*H* and Δ*S* (Figure 1A) in the melting of both type I and type II complexes with the values of their coefficient of determination (*R* ^2^) being 0.9999 and 0.9998, respectively:

2 $$\Delta H=T_{C}\Delta S+\beta$$

where *T*_C_, the compensation temperature, is the slope of the fitting line^5^ and β is the *y*-intercept (Figure 1A). This strong correlation between Δ*H* and Δ*S* is called enthalpy–entropy compensation, which has only been studied and reported in a few cases of starch–lipid complexes,^6^ but also observed in the gelatinization of starch^7^ and protein denaturation.^8^ The enthalpy–entropy compensation (Figure 1A) indicates a compensatory behavior in the melting of starch–lipid complexes. This happens through the increase in Δ*H* to disrupt intermolecular bonds in the starch–lipid complex, which causes an increase in Δ*S* or disorder as the starch and lipid molecules become more disordered.^9–12^

**Figure 1:**
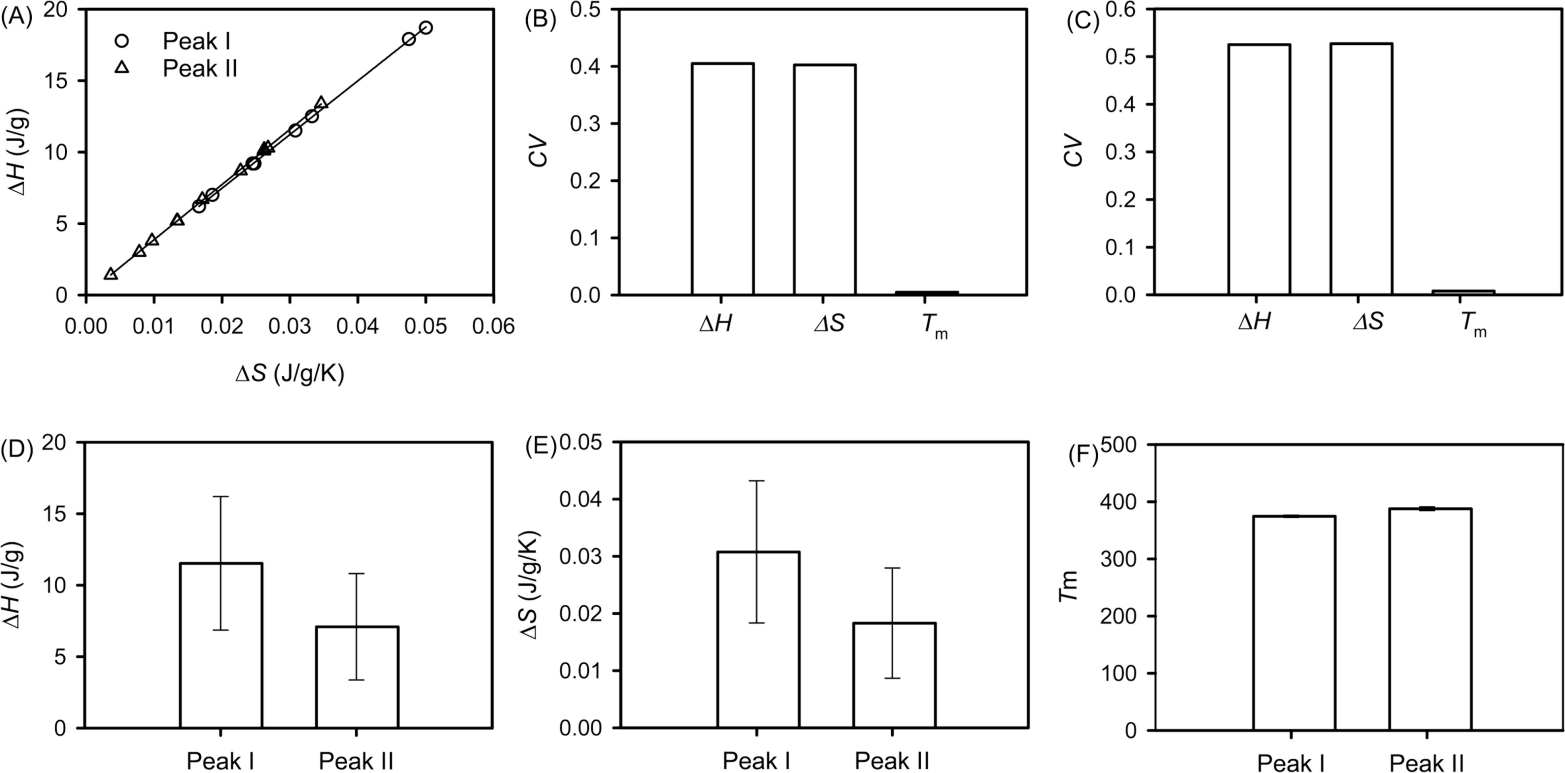
Statistical analysis of the thermodynamic parameters of type I and type II complexes. (A) Enthalpy–entropy compensation for both type I and type II complexes. Coefficients of variation of Δ*H*, Δ*S*, and *T*_m_ for (B) type I complexes and (C) type II complexes. Average values and standard deviations of (D) Δ*H*, (E) Δ*S*, and (F) *T*_m_ for type I and type II complexes. SigmaPlot (version 15, Systat Software Inc., San Jose, CA) was used for graph preparation and statistical analysis.

**Table 1:** DHAMS–FA Complexes Used in the Analysis^a^

sample	peak I	peak II
DHAMS–LA, 70 °C, 10 h	*	
DHAMS–LA, 80 °C, 10 h		*
DHAMS–LA, 90 °C, 10 h		*
DHAMS–LA, 90 °C, 16 h		*
DHAMS–MA, 70 °C, 10 h	*	
DHAMS–MA, 80 °C, 10 h	*	*
DHAMS–MA, 90 °C, 10 h		*
DHAMS–MA, 90 °C, 16 h		*
DHAMS–PA, 70 °C, 10 h	*	
DHAMS–PA, 80 °C, 10 h	*	*
DHAMS–PA, 90 °C, 10 h		*
DHAMS–PA, 90 °C, 16 h		*
DHAMS–SA, 70 °C, 10 h	*	
DHAMS–SA, 80 °C, 10 h	*	
DHAMS–SA, 90 °C, 10 h	*	*
DHAMS–SA, 90 °C, 16 h		*

a Samples marked with an asterisk were included in the analysis. Their *T*_m_ and Δ*H* values are available in the original paper.^1^

The values of *T*_C_ and its standard errors for peak I and peak II are 375.8 ± 1.9 and 385.9 ± 1.8 K, respectively. The results of a Student’s *t*-test between peak I and peak II indicate that the difference in *T*_C_ is statistically significant (Table 2). The degree of freedom (df)^13^ in the *t*-test was calculated as df = (*n*_1_ – 2) + (*n*_2_ – 2), where *n*_1_ and *n*_2_ are the number of DHAMS–FA samples that produced type I and type II crystallites, respectively (*n*_1_ = 8, and *n*_2_ = 11) (Figure 1A). *T*_C_ is recognized as a quantitative measure of the degree of compensation between Δ*H* and Δ*S*.^14,15^ The statistical difference in *T*_C_ between samples with type I and type II crystallites (Table 2) strongly suggests that the melting of DHAMS–FA complexes with different crystalline types follows a unique process.

**Table 2:** Statistical Comparison of the Thermodynamic Parameters between Peak I and Peak II

	*T*_C_	Δ*H*	Δ*S*	*T*_m_
df	15	17	17	17
*t*	–3.7	2.3	2.5	–10.4
*p*	2.0 × 10^–3^	3.4 × 10^–2^	2.4 × 10^–2^	8.7 × 10^–9^

The compensation between Δ*H* and Δ*S* can also be explained by comparing how much variability is present in each thermodynamic parameter. This can be quantitatively determined by comparing the coefficient of variation (CV) for each thermodynamic parameter, using eq 3:

3 $$\mathrm{CV}=\frac{s}{m}$$

where *s* and *m* are the standard deviation and the mean of the samples, respectively.^13^ The CVs of Δ*H* and Δ*S* are more than 70 or 60 times larger than that of *T*_m_ for peak I (Figure 1B) or peak II (Figure 1C), respectively. This suggests that a minimal change in *T*_m_ between the DHAMS–FA samples can result in a significant change in the melting enthalpy and entropy of the DHAMS–FA complexes, which could partly be due to factors such as the chain length of the fatty acids and the complexing times.

To understand the thermodynamic reasons for the high thermal stability of type II complexes, we also compare the thermodynamic parameters between type I and type II complexes. The differences in Δ*H* (Figure 1D), Δ*S* (Figure 1E), and *T*_m_ (Figure 1F) are shown to be statistically significant based on the *p* values (Table 2). Based on these *p* values, *T*_m_ shows the largest difference between type I and type II complexes, showing that *T*_m_ is a key parameter in identifying the crystalline type of a starch–lipid complex. This is in line with the authors’ observations that *T*_m_ is a distinguishing factor in identifying the complex type formed and that type I crystallites are less stable than type II crystallites and therefore more susceptible to amylolysis.^1^ The low thermal stability of type I complexes and their susceptibility to amylolysis can also be explained by the higher value of Δ*S* compared with type II complexes. The average entropy for type I complexes, 0.0308 J g^–1^ K^–1^, was found to be higher than that of type II complexes, 0.0183 J g^–1^ K^–1^ (Figure 1E). This indicates a higher degree of disorder in the melting of type I complexes making type I complexes more susceptible to amylolysis. The analysis of the thermodynamic parameters shows why Δ*S* should also be included in explaining the variation of *T*_m_ and not Δ*H* alone.

For starch–lipid complexes to melt spontaneously or for the process to be thermodynamically favorable, the Gibbs free energy (Δ*G*) must be negative,^16^ which depends not only on Δ*H* but also on Δ*S* as shown in eq 4:

4 ΔG=ΔH−TΔS

If Δ*S* is high, it compensates for a high Δ*H*, which makes the melting of the starch–lipid complex occur more easily or favorably.^17^ Therefore, even though the type I complexes have a higher average Δ*H* of 11.525 J/g than type II complexes with an average Δ*H* of 7.091 J/g, which may indicate a higher stability of type I complexes, they also have the highest average Δ*S* of 0.0308 J g^–1^ K^–1^ compared to type II complexes with an average Δ*S* of 0.0183 J g^–1^ K^–1^, indicating that type II complexes rather possess a higher stability. This shows that explaining their stability in terms of Δ*H* alone can be misleading. The high Δ*S* in type I complexes compensates for its high Δ*H*, which makes the melting of type I complexes occur easily with a lower average *T*_m_ (374.51 K) compared to that of type II complexes (387.69 K). Therefore, Δ*S* helps to explain why in some complex types, melting may occur more easily despite a high Δ*H*, because the increase in Δ*S* leads to an increase in the disorder of the system, driving the complex to transition into a melted state. This makes Δ*S* a key factor in explaining the melting of complexes, such as starch–lipid complexes.

While our analysis identified numerical values of Δ*S* in the phase transition of starch–lipid complexes, it would provide more insights into the system if the magnitude of Δ*S* could be related to structural and/or physical properties of the complex. For example, in a recent paper, Δ*S* was used to predict the feasibility of removal of heavy metals using the oceanic abundance of metals.^18^ We therefore examined the relationship between the properties of the starch–lipid complexes and Δ*S* and how that relationship could explain the differences in type I and type II complexes. We found moderately strong relationships between Δ*S* and the X-ray diffraction (XRD) peaks compared to the other properties (Figure 2). Interestingly, while type I complexes had a direct positive relationship with Δ*S* for the diffraction peaks, type II complexes had an inverse relationship with Δ*S*. The 7.5°, 13.0°, and 19.9° diffraction peaks reveal information about the crystalline structure of the starch–lipid complexes.^19^ The strong positive relationship with the diffraction peaks suggests that as the crystallinity increases so does the Δ*S* for type I complexes. For type II complexes, the negative relationship with the diffraction peaks suggests that as the crystallinity increases, Δ*S* decreases. The opposite directions in Δ*S* could, therefore, be explained by unique crystalline structures in the two complex types. This further explains the statistical difference in *T*_C_ between type I and type II complexes, that is, how their melting follows a unique process.

**Figure 2:**
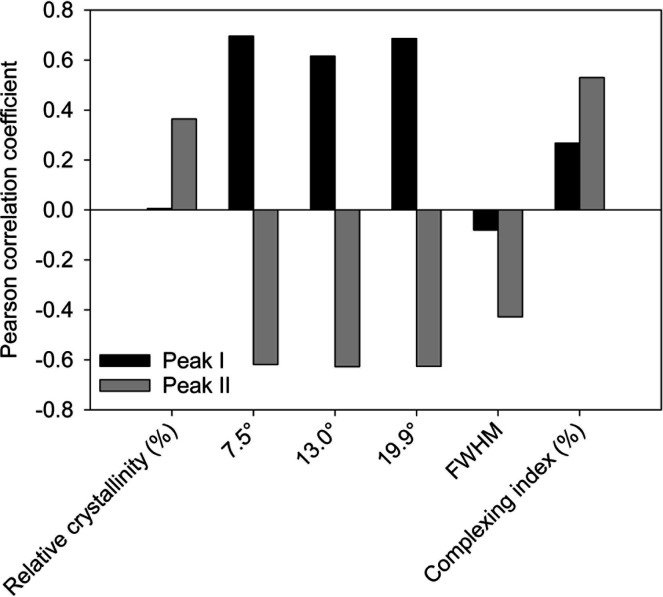
Correlation between Δ*S* and relative crystallinity, the full width at half-maximum (fwhm) of the XRD peaks, the fwhm of the Raman band at 480 cm^–1^, and the complexing index for both type I and type II complexes.

In conclusion, the melting of starch–lipid complexes, for both type I and type II complexes, exhibits enthalpy–entropy compensation. Statistical analysis suggests that *T*_m_ is a key parameter in identifying the crystalline type of complexes and Δ*S* plays a role in the high thermal stability of type II complexes and in understanding why some complexes melt more easily than others. This can serve as a guide for the food industry in controlling the melting behavior of starch–lipid complexes to enhance product quality.
